# Evaluation of Oxasqualenoids from the Red Alga *Laurencia viridis* against *Acanthamoeba*

**DOI:** 10.3390/md17070420

**Published:** 2019-07-19

**Authors:** Jacob Lorenzo-Morales, Ana R. Díaz-Marrero, Francisco Cen-Pacheco, Ines Sifaoui, María Reyes-Batlle, María L. Souto, Antonio Hernández Daranas, José E. Piñero, José J. Fernández

**Affiliations:** 1 Instituto Universitario de Enfermedades Tropicales y Salud Pública de Islas Canarias, Universidad de La Laguna, Avda. Astrofísico F. Sánchez s/n, 38206 La Laguna, Tenerife, Spain; 2 Instituto Universitario de Bio-Orgánica Antonio González (IUBO AG), Centro de Investigaciones Biomédicas de Canarias (CIBICAN), Universidad de La Laguna (ULL), Avda. Astrofísico F. Sánchez, 2, 38206 La Laguna, Tenerife, Spain; 3 Facultad de Bioanálisis, Campus-Veracruz, Universidad Veracruzana, Veracruz 91700, Mexico; 4 Departamento de Química Orgánica, Universidad de La Laguna (ULL), Avda. Astrofísico F. Sánchez, 2, 38206 La Laguna, Tenerife, Spain; 5 Instituto de Productos Naturales y Agrobiología (IPNA), Consejo Superior de Investigaciones Científicas (CSIC), Avenida Astrofísico Francisco Sánchez 2, 38206 Tenerife, Spain

**Keywords:** oxasqualenoid, marine natural product, *Acanthamoeba*, triterpene, *Laurencia*, dehydrothyrsiferol

## Abstract

*Acanthamoeba* genus is a widely distributed and opportunistic parasite with increasing importance worldwide as an emerging pathogen in the past decades. This protozoan has an active trophozoite stage, a cyst stage, and is dormant and very resistant. It can cause *Acanthamoeba* keratitis, an ocular sight-threatening disease, and granulomatous amoebic encephalitis, a chronic, very fatal brain pathology. In this study, the amoebicidal activity of sixteen *Laurencia* oxasqualenoid metabolites and semisynthetic derivatives were tested against *Acanthamoeba castellanii* Neff. The results obtained point out that iubol (**3**) and dehydrothyrsiferol (**1**) possess potent activities, with IC_50_ values of 5.30 and 12.83 µM, respectively. The hydroxylated congeners thyrsiferol (**2**) and 22-hydroxydehydrothyrsiferol (**4**), active in the same value range at IC_50_ 13.97 and 17.00 µM, are not toxic against murine macrophages; thus, they are solid candidates for the development of new amoebicidal therapies.

## 1. Introduction

*Acanthamoeba* spp. (family Acanthamoebidae, order Centramoebidae) [1] are opportunistic free-living amoebae (FLA) that are ubiquitous in nature and widely distributed in different environments such as seawater, soil, lakes, contact lenses, air-conditioning units, as well as hospitals [2,3]. *Acanthamoeba* spp. are opportunistic pathogens to humans and other animals [4,5]. *Acanthamoeba* keratitis (AK) and granulomatous amoebic encephalitis (GAE) are the main infections caused by *Acanthamoeba* spp. Moreover, it is also a causative agent of nasopharyngeal and cutaneous infections [6,7]. AK is a sight-threatening infection that affects the cornea. In the case of AK, the parasite adheres to the corneal surface, binding to glycoproteins on the epithelium. This allows the release of enzymes and toxins, like protease, phospholipases, and cytopathic factors including mannose-induced protein, leading to corneal epithelial destruction. Thus, *Acanthamoeba* is able to invade the stroma and secrete collagenolytic factors, dissolving the stromal matrix, which results in an inflammatory response that leads to corneal cell death and keratitis [8,9]. It can provoke epithelial defects, lid edema, and even pain with photophobia. Although the biggest risk to develop AK is by wearing contact lenses (more than 80% of the cases), since it facilitates adhesion to the corneal surface, people who do not wear them can also suffer from this infection. Diagnosis of AK is complicated and may be easily misdiagnosed as *Herpes simplex* keratitis [10,11]. The most commonly used drugs for AK treatment are biguanides i.e., polyhexamethylene biguanide (PHMB), or chlorhexidine digluconate along with a diamidine such as propamidine isethionate or hexamidine. The treatments are often lengthy and require close medical supervision. Furthermore, the cyst stage further complicates the treatment of *Acanthamoeba* infections [12]. In the case of suspected infection or coinfection, antibiotics like neomycin or chloramphenicol should be added to the regimen, not only to prevent bacterial infection but also to eliminate the food source for the parasite [2].

Marine organisms are the source of structurally diverse and biologically active secondary metabolites, which have inspired the development of several drugs to treat human diseases, despite none being in the therapeutic area of antiparasitics [13]. Among marine organisms, seaweeds of *Laurencia* species are prolific producers of sesquiterpenes, diterpenes, triterpenes, and C_15_ acetogenins, and they have been tested for a wide range of biological activities—cytotoxic, antiviral, anti-inflammatory—and against parasites and their vectors [14,15,16].

In a previous work, we reported on the amoebicidal activity of the natural sesquiterpenes, α-bromocuparane and α-isobromocuparane, isolated from *Laurencia johnstonii*, including the most active compound, 3α-bromojohnstane, a halogenated semisynthetic derivative that showed an IC_50_ of 41.51 µM against *Acanthamoeba castellanii* Neff [16].

**Figure 1:**
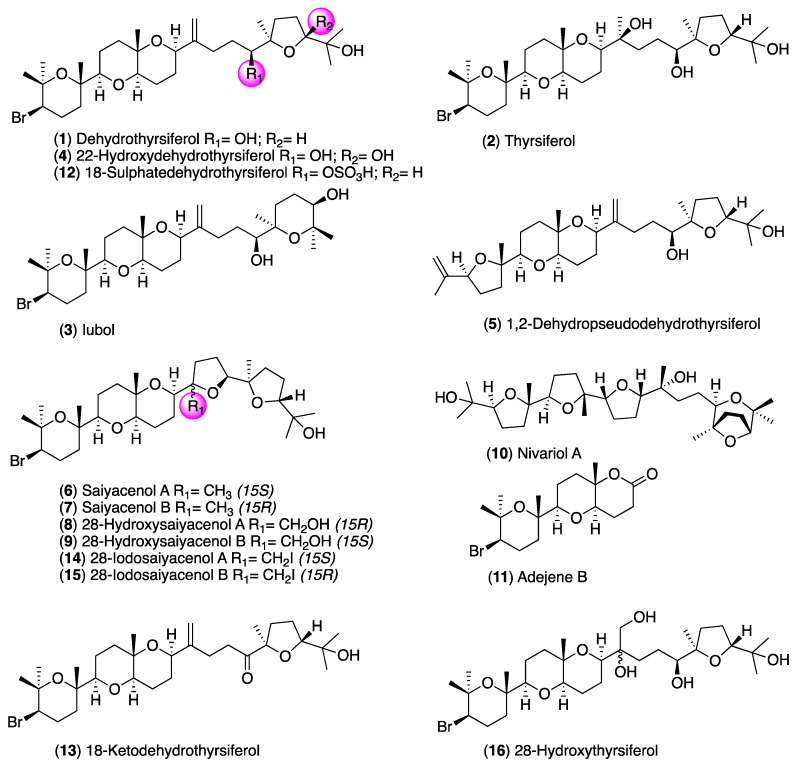
Structures of natural oxasqualenoids (**1**–**11**) obtained from the red alga *Laurencia viridis* and semisynthetic substances (**12**–**16**) obtained from the main metabolite dehydrothyrsiferol (**1**).

In this work, oxasquealenoids—including dehydrothyrsiferol (DT) (**1**) [17]; thyrsiferol (**2**) [18]; iubol (**3**) [19]; 22-hydroxydehydrothyrsiferol (**4**) [19]; 1,2-dehydropseudodehydrothyrsiferol (**5**) [19]; saiyacenols A and B (**6** and **7**) [20]; 28-hydroxysaiyacenol A and B (**8** and **9**) [21]; nivariol A (**10**) [22]; and the truncated metabolite adejene B (**11**) [23]—were isolated from the marine alga *Laurencia viridis* (Figure 1). The substances obtained by semi-synthetic methods—including 18-sulphatedehydrothyrsiferol (**12**); 18-ketodehydrothyrsiferol (**13**); 28-iodosaiyacenols A and B (**14** and **15**); and 28-hydroxythyrsiferol (**16**)—were evaluated in vitro against *Acanthamoeba castellanii* Neff, and a structure–activity relationship was also established.

## 2. Results and Discussion

A discrete collection of natural oxasquealenoids (**1**–**11**) obtained from specimens of *Laurencia viridis* and semisynthetic DT derivatives (**12**–**16**) were selected to screen their in vitro activities against *Acanthamoeba castellanii* Neff. The results of the antiamoeboid effect are indicated in Table 1. All tested compounds showed activity against *A. castellanii* trophozoites. Compound **3**, iubol, was the most active compound, with an IC_50_ of 5.30 ± 0.87 µM, comparable to that of the reference drug chlorhexidine, followed by DT (**1**) with a value of IC_50_ of 12.83 ± 1.38 µM. A similar range of activity was observed for thyrsiferol (**2**) and 22-hydroxydehydrothyrsiferol (**4**) with IC_50_ values of 13.97 ± 1.57 µM and 17.00 ± 4.57 µM, respectively. In fact, ANOVA showed a nonsignificant difference (*p* < 0.05) in the activity effect between compounds (**2**) and (**4**).

**Figure 2:**
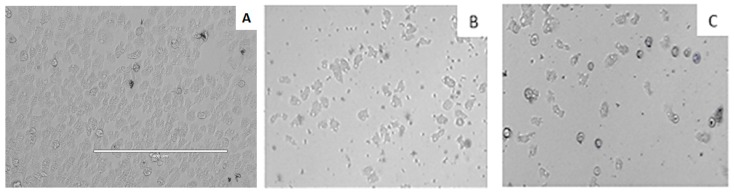
Growth inhibition of *Acanthamoeba castellanii* trophozoites. Negative Control (**A**), iubol (**3**) (**B**), and DT (**1**) (**C**) using a concentration of 42.5 µM (25 µg/mL). All images (10×) are representative of the population of treated amoeba and are based on the live cell imaging microscope EVOS FL cell imaging system.

Figure 2 shows the inhibitory effect of iubol (**3**) and DT (**1**) on the growth of *A. castellanii* trophozoites at a concentration of 42.5 µM (25 µg/mL). Compared with the negative control, many of the treated cells were dead and others lost their characteristic cellular shape, highlighting their trophocidal activity.

The toxicities of compounds **1**–**16** were determined in vitro against murine macrophages (J774A.1). Iubol (**3**), the most active compound, turned out to be the most cytotoxic with a CC_50_ of 7.72 ± 0.22 µM. On the other hand, DT (**1**) was less toxic than **3** with a CC_50_ of 28.77 ± 3.10 µM, as well as 18-ketodehydrothyrsiferol (**13**), 28-iodosaiyacenol A (**14**), and saiyacenol A (**6**) with CC_50_ of 23.37 ± 1.76, 29.45 ± 0.20, and 59.91 ± 8.50 µM, respectively. The rest of the molecules showed CC_50_ values higher than 1000 µM; therefore, due to its cytotoxicity levels, iubol (**3**) was excluded from further analyses.

**Figure 3:**
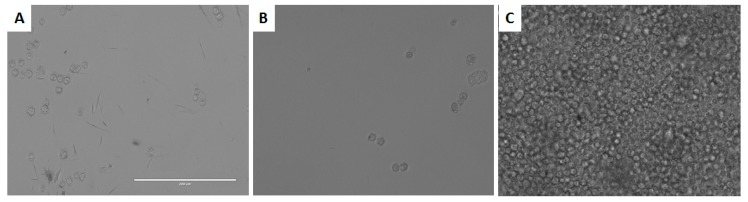
Effect of dehydrothyrsiferol (DT) on the cyst of *A. castellanii* Neff at 170 µM (**A**), 85 µM (**B**), and negative control (**C**). All images (20×) are representative of the population of treated amoeba and are based on the live cell imaging microscope EVOS FL cell imaging system.

As it could be observed in Figure 3, DT exhibited cysticidal activity against *A. castellanii* Neff, with an IC_50_ of 39.27 ± 0.14 µM. DT was tested against the trophozoite stage of two clinical strains, *Acanthamoeba griffini* and *Acanthamoeba polyphaga*, showing IC_50_ values of 56.66 ± 2.09 and 73.44 ± 4.02 µM, respectively.

Consequently, considering the preliminary screening values, the major metabolite DT (**1**) was selected as the candidate molecule to continue the studies on the mode of action on *A. castellanii* Neff. Drugs that do not produce necrotic cell death is the main focus in the search for new therapies. Thus, programmed cell death or apoptosis-like processes in protozoan parasites are the subject of study and include several morphological events such as chromatin condensation, nuclear DNA fragmentation, or a decrease of cellular ATP level, among others [24]. At this stage, the objective was to understand the mechanism of action of DT and to evaluate changes in mitochondrial membrane potential and in the ATP production.

**Figure 4:**
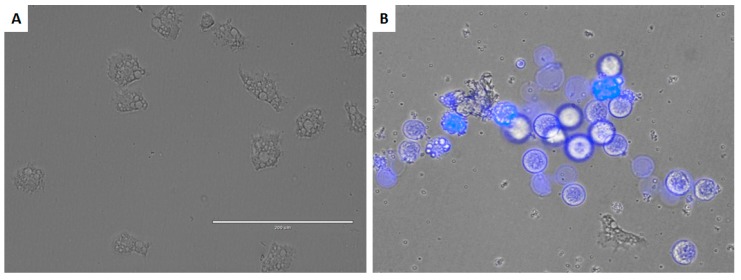
Effect of IC_90_ of DT for 24 h on the chromatin condensation (**B**) (overlay Hoechst 33342/propidium iodide double stain) and negative control (**A**). All images (20×) are representative of the population of treated amoeba and are based on the live cell imaging microscope EVOS FL cell imaging system.

DT-treated cells stained positive in the double-stain assay. When double staining was performed, it was observed that the tested drug at the IC_90_ concentration could induce chromatin condensation, as proven by the bright blue nuclei stain shown in Figure 4.

**Figure 5:**
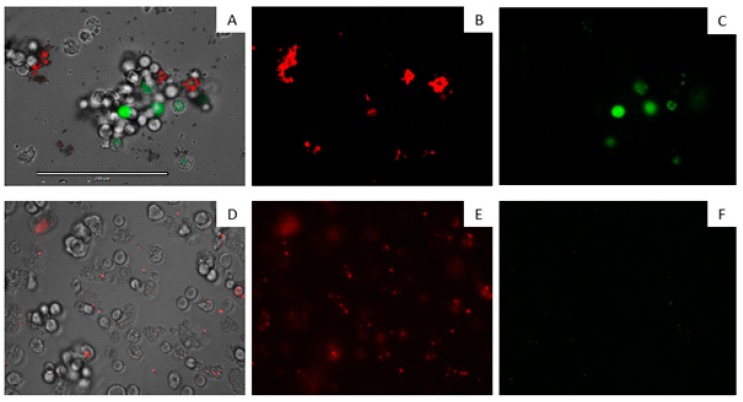
Effects on the mitochondrial potential in cells treated with the IC_90_ of DT for 24 h (**A**)–(**C**). Due to collapse of mitochondrial potential, the JC-1 dye remained in the cytoplasm in its monomeric form, green fluorescence (**C**). In negative controls (**D**)–(**F**), JC-1 dye accumulates in the mitochondria of healthy cells as aggregates (red fluorescence) (**E**). All images (20×) are representative of the population of treated amoeba and are based on the Live Cell Imaging Microscope EVOS FL Cell Imaging System.

DT induced mitochondrial malfunction in the parasites since the ATP levels and mitochondrial membrane potential collapsed after treatment with the compound. This observation is supported in Figure 5, which shows that cells treated with DT emitted a higher green fluorescence (C) than the negative control (F). This indicates the presence of JC-1 (5,5′,6,6′-tetrachloro-1,1′,3,3′-tetraethyl-imidacarbocyanine iodide) monomers, which reflects a decrease of the mitochondrial membrane potential. It is also possible to observe that, in treated cells, there was a lower red fluorescence (B) than in control (E). In this case, the mitochondrial potential was not altered, allowing JC-1 to aggregate.

**Figure 6:**
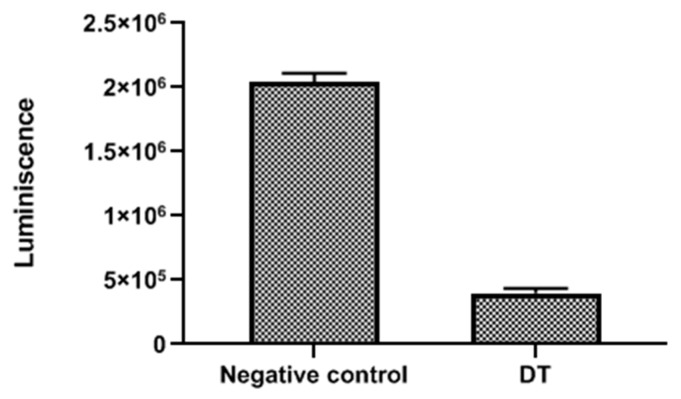
ATP measurement assay with negative control and DT.

The mitochondrial damage was documented by measuring the ATP level generated in 24 h. We found that the IC_90_ produced a pronounced decrease in the total ATP level by 80.48%, in comparison to the negative control, meaning that the cells only maintained 19.52% of the ATP level after treatment with DT (Figure 6).

**Figure 7:**
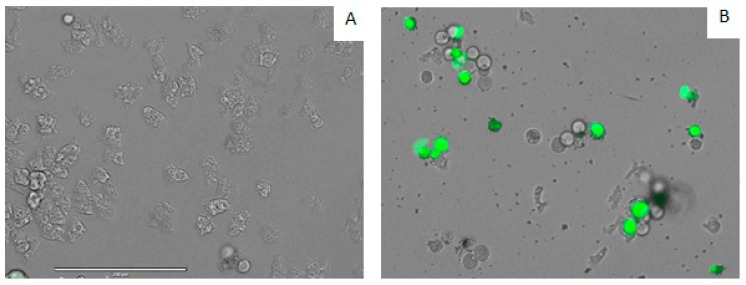
Permeabilization of *A. castellanii* Neff to *SYTOX*^®^ Green dye caused by addition of IC_90_ of DT (**B**) compared with the negative control (**A**). All Images (20×) are representative of the population of treated amoeba and are based on the Live Cell Imaging Microscope EVOS FL Cell Imaging System.

DT caused plasma membrane permeability in treated cells. Amoebae treated with the IC_90_ of DT showed plasmatic membrane damage after 24 h of incubation. Moreover, it is also important to mention that even though the membrane was damaged, cell integrity was maintained, as confirmed in Figure 7.

**Figure 8:**
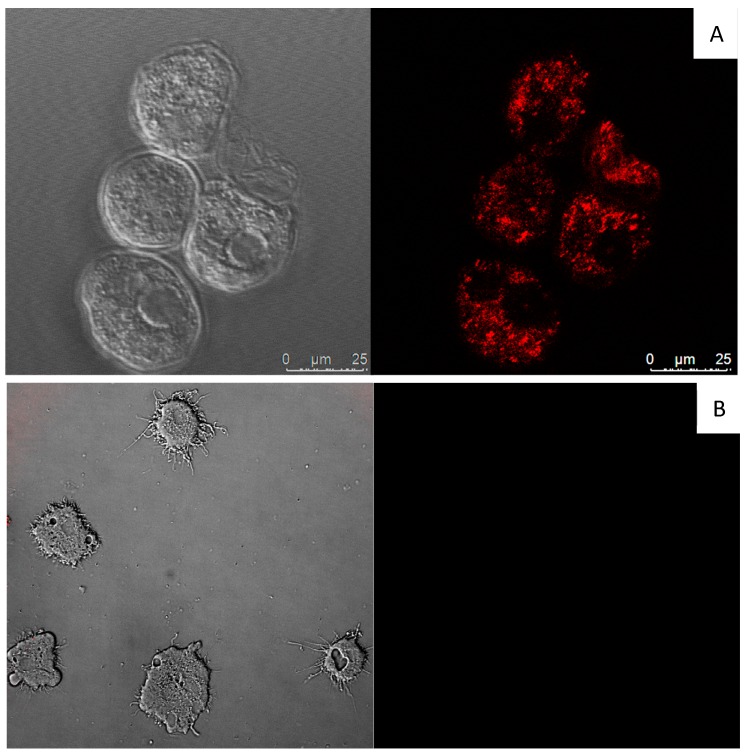
Increased levels of reactive oxygen species (ROS) in *A. castellanii* Neff observed after incubation with the DT IC_90_ for 24 h (**A**) compared with the negative control (**B**). Images (63×) are representative of the cell population in the performed experiments. Cells were observed in a Leica TSC SPE confocal microscope equipped with inverted optics.

DT increases reactive oxygen species (ROS) levels in *A. castellanii* Neff. Thus, DT-treated amoebae caused increased levels of ROS when treated with the IC_90_ after 24 h of incubation (Figure 8).

**Figure 9:**
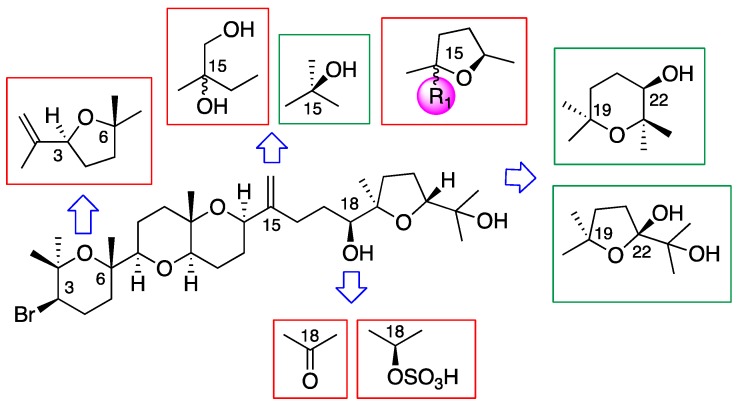
Structure–activity relationship (SAR) in Laurencia oxasqualenoids against *A. castellanii* Neff.

Assuming the major compound in the alga, dehydrothyrsiferol (**1**), as a model molecule for a structure–activity relationship (SAR) discussion, the main structural modifications that have been analyzed are summarized in Figure 9. Thus, the fragments that imply a loss of activity are embedded in red color, whereas the green color indicates those molecular features that either improved the activity or that, keeping it within the same range, significantly decreased the toxicity in macrophages. Simplifications in the size of the molecule, as is the case of adejene B (**11**) (fragment C-1-C-14), and bromine losses as for 1,2-dehydropseudodehydrothyrsiferol (**5**) or nivariol A (**10**) lead to less active substances. In the same way, chemical modifications were carried out on DT (**1**), including oxidation and sulfonation at C-18 as well as the diol formation at C-15-C-28, which led to substances with very low activities, with 28-hydroxythyrsiferol (**16**) being the least active compound of this series. All molecules that introduced an additional tetrahydrofuran ring between C-15 and C-18 did not improve their activity with respect to DT (**1**).

On the other hand, modification of the tetrahydrofuran terminal ring between C-19 and C-22 by a tetrahydropyran ring between C-19 and C-23 led us to the most active compound, iubol (**3**), which was also the most toxic against murine macrophage J774A.1. Undoubtedly, the two specific modifications that showed the best results were both the introduction of a hydroxyl group, at C-15 as in thyrsiferol (**2**) and at C-22 as in 22-hydroxydehydrothyrsiferol (**4**). Thus, **2** and **4** possess a relevant biological activity against *A. castellanii* Neff, with IC_50_ of 13.97 ± 1.57 and 17.00 ± 4.57 µM, respectively. This was combined with a very low toxicity against murine macrophage J774A.1 (CC_50_ < 50), and these were the most important chemical modifications on the leader molecule to improve both its antiparasitic potency and mitigate its lateral toxic effects.

## 3. Materials and Methods

### 3.1. General Methods

All reagents were commercially available and used as received. All solvents were dried and distilled under argon immediately prior to use or stored appropriately. Flash chromatography was performed with silica gel (230−400 mesh) as the stationary phase and mixtures of *n*-hexane and ethyl acetate (EtOAc), in different proportions given in each case, as the mobile phase. Melting points were determined on a Büchi B-540 model. Optical rotations were determined on a PerkinElmer 343 polarimeter using a sodium lamp operating at 589 nm. IR spectra were measured on a Bruker IFS 66 spectrometer (Ettlingen, Germany) using a methanolic solution over a NaCl disk or neat. NMR spectra were performed on Bruker Avance 400, 500, or 600 instruments (Bruker Biospin, Falländen, Switzerland) at 300 K, and coupling constants are given in Hz. COSY, 1D/2D TOCSY, HSQC, HMBC, and ROESY experiments were performed using standard pulse sequences. Phase-sensitive ROESY spectra were measured using a mixing time of 500 ms. Data were processed using Topspin or MestRe software (Mestrelab Research, S.L., Santiago de Compostela, Spain). Mass spectra were recorded on an LCT Premier XE Micromass spectrometer using electrospray ionization. EnSpire^®^ Multimode Reader (Perkin Elmer, Waltham, MA, USA) used absorbance values of Alamar Blue^®^ reagent (Bio-Rad Laboratories, Oxford, UK). Thin Layer Chormatography (TLC) was performed on AL Si gel. TLC plates were visualized by UV light (254 nm) and by adding a phosphomolybdic acid solution 10% (*w*/*v*) in MeOH or a vanillin solution (6 g of vanillin, 450 mL of ethanol (EtOH), 40 mL of EtOAc, and 30 mL of H_2_SO_4_).

### 3.2. Isolation of Laurencia Metabolites

All metabolites were isolated from biomass obtained from several annual harvestings between April 2013–April 2018 of the red alga *Laurencia viridis* at the Paraiso Floral, Tenerife, Canary Islands (28°07′12″ N, 16°46′45″ W). Dried specimens from the sterile plants, sporophytes, and gametophytes were filed at TFC Phyc 7180 (Herbario de la Universidad de La Laguna, Departamento de Biologia Vegetal, Botánica, Tenerife, Spain). In all cases, the individual biomasses of *L. viridis* were extracted with chloroform:methanol (CHCl_3_:MeOH) (1:1) at room temperature (rt), and a dark green, viscous oil was obtained after concentration under reduced pressure. The extracts were first chromatographed using Sephadex LH-20 (7 × 50 cm) using CH_2_Cl_2_:MeOH (1:1) as the mobile phase. The enriched polyether fractions were subsequently processed on a silica gel column (7 × 50 cm) using a linear gradient of *n*-hexane:EtOAc (80:20−20:80), and the oxasqualenoids fractions were monitored by NMR. Next, medium-pressure chromatography was done on Lobar LiChroprep Si-60 using CH_2_Cl_2_:acetone (8:2) at 1 mL/min. Final purification was done on an HPLC with a μ-Porasil column using *n*-hexane:EtOAc:MeOH, 18:15:5. Using this protocol, the following pure powdery compounds were obtained for the biological assays (% dry weight): dehydrothyrsiferol (DT) (**1**), (0.093%); thyrsiferol (**2**), (0.00017%); iubol (**3**), (0.00018%); 22-hydroxydehydrothyrsiferol (**4**), (0.000041%); 1,2 dehydropseudodehydrothyrsiferol (**5**), (0.00029%); saiyacenol A(**6**), (0.00018%); saiyacenol B (**7**), (0.00019%); 28-hydroxysaiyacenol A (**8**), (0.00018%); 28-hydroxysaiyacenol B (**9**), (0.00019%); nivariol A (**10**), (0.00013%); and the truncated metabolite adejene B (**11**), (0.00019%).

### 3.3. Chemical Transformations of Dehydrothyrsiferol (***1***)

#### 3.3.1. Preparation of 18-sulphatedehydrothyrsiferol (**12**)

An SO_3_-pyridine complex (55.7 mg, 0.35 mmol) was added to a solution of DT (**1**) (20 mg, 0.034 mmol) in pyridine (5 mL) at room temperature. After stirring at 80 °C for 3 h, the reaction mixture was quenched with MeOH. Removal of the solvent gave a residue, which was purified by Sephadex LH-20 column (CHCl_3_:MeOH 1:1) followed by HPLC chromatography (XTerra column, MeOH:H_2_O 17:3 as eluent and 1.5 mL/min flow rate) that afforded 18-sulphatedehydrothyrsiferol (**12**) (14 mg, powder) (yield: 62%). ^1^H NMR (600 MHz, CDCl_3_): δ 1.18 (s, H_3_-24), 1.18 (s, H_3_-26), 1.18 (s H_3_-30), 1.21 (s, H_3_-29), 1.21 (s H_3_-27), 1.26 (s, H_3_-1), 1.39 (s, H_3_-25), 1.45 (H-8), 1.50 (H-9), 1.50 (H-21), 1.54 (H-5), 1.56 (H- 12), 1.57 (H-20), 1.72 (H’-8), 1.76 (H’-12), 1.77 (H’-9), 1.80 (H’-21), 1.81 (H’-5), 1.82 (H-13), 1.99 (H’-13), 2.10 (H-4), 2.23 (H’-4), 2.23 (H- 16), 2.27 (H’-20), 2.40 (H’-16), 3.07 (dd, *J* 1⁄4 2.1, 11.1 Hz, H-7), 3.41 (dd, *J* 1⁄4 5.7, 11.3 Hz, H-11), 3.89 (dd, *J* 1⁄4 4.1, 12.3 Hz, H-3), 4.06 (H- 22), 4.23 (dd, *J* 1⁄4 4.0, 8.3 Hz, H-14), 4.54 (H-18), 4.83 (bs, H-28), 5.01 (bs, H’-28). ^13^C NMR (150 MHz, CDCl_3_): δ 19.6 (C-27), 20.3 (C-26), 21.3 (C-24), 21.3 (C-30), 21.8 (C-12), 23.1 (C-8), 23.7 (C-25), 25.3 (C- 29), 26.6 (C-13), 28.1 (C-21), 28.4 (C-4), 28.4 (C-16), 30.9 (C-1), 30.9 (C-17), 31.5 (C-20), 37.4 (C-5), 38.7 (C-9), 59.0 (C-3), 72.3 (C-23), 72.5 (C-14), 72.8 (C-10), 74.2 (C-6), 74.8 (C-2), 78.7 (C-11), 83.6 (C- 18), 85.4 (C-19), 86.5 (C-7), 87.1 (C-22), 109.2 (C-28), 151.4 (C-15).

#### 3.3.2. Chemical Transformation of Dehydrothyrsiferol (**1**) into 18-Ketodehydrothyrsiferol (**13**)

Pyridinium chlorochromate (2.5 mg, 0.011 mmol) was added to a solution of DT (1) (5 mg, 0.008 mmol) in CH_2_Cl_2_ (0.7 mL). The resulting mixture was stirred for 3 h at room temperature (27 °C). Afterwards, the solution was filtered and concentrated to give a solid residue that was chromatographed using HPLC (XTerra column, *n*-hex:AcOEt 8:2, flow rate 1 mL/min), affording 18-keto-dehydrothyrsiferol (1.4 mg, powder) (yield 28%). 18-Keto-dehydrothyrsiferol: ${\left[\alpha \right]}_{D}^{25}$ + 27 (c 0.14, CHCl_3_); ^1^H NMR: (CDCl_3_; 500 MHz) δ 1.15 (s, H_3_-24), 1.20 (s, H_3_-26), 1.22 (s, H_3_-30), 1.25 (s, H_3_-25), 1.27 (s, H_3_-1), 1.34 (s, H_3_-29), 1.40 (s, H_3_-25), 1.44 (Hα-9), 1.49 (Hβ-8), 1.52 (Hα-5), 1.60 (Hβ-12), 1.73 (H-20), 1.76 (Hα-8), 1.77 (Hβ-9), 1.80 (Hβ-5), 1.81 (Hα-12), 1.84 (H2-21), 1.86 (Hβ-13), 2.11 (Hα-13 and H’-20), 2.12 (H-16), 2.13 (Hα-4), 2.26 (Hβ-4), 2.40 (H’-16), 2.70 (ddd, *J* = 5.4, 9.1, 18.0 Hz, H-17), 2.93 (ddd, *J* = 5.8, 9.5, 18.0 Hz, H’-17), 3.08 (dd, *J* = 2.5, 11.0 Hz, H-7), 3.42 (dd, *J* = 5.7, 11.3 Hz, H-11), 3.77 (dd, *J* = 6.0, 9.9 Hz, H-22), 3.89 (dd, *J* = 4.1, 12.6 Hz, H-3), 4.29 (dd, *J* = 4.0, 8.1 Hz, H-14), 4.86 (bs, H-28), 5.03 (bs, H’-28). ^13^C NMR: (CDCl_3_; 125 MHz) δ 19.3 (C-27), 20.2 (C-26), 21.9 (C-12), 23.0 (C-8), 23.5 (C-25), 24.3 (C-24), 24.5 (C-29), 26.3 (C-21), 26.6 (C-13), 27.7 (C-30), 28.0 (C-4), 29.8 (C-16), 31.1 (C-1), 35.0 (C-20), 35.5 (C-17), 37.1 (C-5), 38.9 (C-9), 59.1 (C-3), 70.5 (C-23), 72.5 (C-14), 72.7 (C-10), 74.3 (C-6), 75.0 (C-2), 78.8 (C-11), 86.7 (C-7), 87.6 (C-22), 89.0 (C-19), 109.9 (C-28), 151.4 (C-15), 215.3 (C-18).

#### 3.3.3. Chemical Transformations of Dehydrothyrsiferol (**1**) into 28-Iodosaiyacenol A and B (**14** and **15**)

Iodine (17.3 mg, 68.16 mmol) and sodium bicarbonate (5.72 mg, 68.16 mmol) were added sequentially to a stirred solution of DT (**1**) (20 mg, 0.034 mmol) in dry CH_2_Cl_2_ (1 mL), and the resulting mixture was stirred for 5 h at room temperature. The solution was filtered and concentrated under reduced pressure to give a mixture of isomeric iododerivatives of saiyacenol A and B. Afterwards, the reaction mixture was then concentrated in vacuo and purified by HPLC (μ-Porasil column, *n*-hexane/acetone 9:1, flow rate 1 mL/min), affording 28-iodosaiyacenol A (**14**, 5.5 mg, powder) (yield 22.6%) and B (**15**, 10.6 mg, powder) (yield 43.0%).

#### 3.3.4. Synthesis of 15,28 Diol-Compounds (**16**)

A catalytic OsO_4_ and dehydrothyrsiferol (**1**) (20 mg, 0.034 mmol) in 2 mL acetone were added to N-methylmorpholine-N-oxide (NMO, 24 mg, 0.205 mmol) in acetone:H_2_O mixture (4:1; 3 mL). The reaction was stirred for 24 h at room temperature. The excess OsO_4_ was quenched by addition of NaHCO_3_ (2 mg, 0.024 mmol), and the solvent mixture was removed in vacuo. The residue was purified by Sephadex LH-20 column (CHCl_3_:MeOH 1:1) followed by an HPLC (μ-Porasil column; *n*-Hex:AcOEt:MeOH 14:5:1 as eluent; 0.75 mL/min flow rate) to give the mixture of 28-hydroxytyrsiferol (15 mg, powder) (yield 70%). 28-hydroxytyrsiferol: ^1^H NMR (600 MHz, CDCl_3_): δ 1.14 (s, H_3_-24), 1.16 (s, H_3_-29), 1.20 (s, H_3_- 26), 1.21 (s, H_3_-30), 1.23 (s, H_3_-27), 1.28 (s, H_3_-1), 1.35 (H-17), 1.40 (s, H_3_-25), 1.43 (H-8), 1.52 (H-16), 1.53 (H-9), 1.53 (H-12), 1.54 (H-5), 1.60 (H-20), 1.65 (H0 -17), 1.75 (H’-16), 1.76 (H’-8), 1.78 (H’-9), 1.79 (H-13), 1.81 (H’-5), 1.84 (H_2_-21), 1.92 (H’-12), 1.96 (H’-13), 2.11 (H-4), 2.11 (H’-20), 2.25 (H’-4), 3.07 (dd, *J* 1⁄4 1.9, 11.4 Hz, H-7), 3.45 (d, *J* 1⁄4 11.3, H-28), 3.48 (dd, *J* 1⁄4 1.3, 9.7 Hz, H-18), 3.61 (dd, *J* 1⁄4 8.1, 12.5 Hz, H-11), 3.73 (d, *J* 1⁄4 11.3 Hz, H’-28), 3.77 (dd, *J* 1⁄4 5.8, 10.1 Hz, H-22), 3.90 (dd, *J* 1⁄4 4.0, 12.3 Hz, H-3), 3.96 (dd, *J* 1⁄4 2.5, 12.6 Hz, H- 14). ^13^C NMR (150 MHz, CDCl_3_) δ 20.1 (C-26), 21.0 (C-12), 21.1 (C-13), 21.4 (C-27), 23.0 (C-8), 23.6 (C-25), 23.9 (C-24), 23.9 (C-29), 24.8 (C- 17), 26.3 (C-21), 27.7 (C-30), 28.3 (C-4), 30.6 (C-16), 31.0 (C-1), 31.7 (C-20), 37.0 (C-5), 38.5 (C-9), 59.0 (C-3), 65.3 (C-28), 70.7 (C-23), 72.7 (C-10), 74.4 (C-6), 75.0 (C-2), 75.3 (C-15), 75.9 (C-11), 76.1 (C- 14), 77.5 (C-18), 86.1 (C-19), 86.7 (C-7), 87.6 (C-22).

#### 3.3.5. Preparation of Synthetic 28-Hydroxysaiyacenol A and B (**8** and **9**)

Due to the small amount of sample available from natural source of compounds **8** and **9**, these were also prepared from DT (**1**). m-Chloroperbenzoic acid (1.5 equiv) was added into a CH_2_Cl_2_ (0.5 mL) solution containing 1.5 mg of DT (**1**) (0.0025 mmol). The resulting mixture was stirred for 3 h at rt. Afterward, the solution was filtered and concentrated to give a solid residue, which was chromatographed using HPLC (μ-Porasil column, *n*-hexane/acetone, 7:3, flow rate 1 mL/min), affording 0.6 mg of 28-hydroxysaiyacenol A (**8**) (powder, yield 40%) and 0.8 mg of 28-hydroxysaiyacenol B (**9**) (powder, yield 53%). All spectroscopic data matched with those of natural compounds.

### 3.4. Cell Strains and Chemical Inhibitors

The antiamoeboid activity of the compounds was evaluated against *Acanthamoeba castellanii* Neff (ATCC 30010) type strain from the American Type Culture Collection. This strain was axenically incubated in PYG medium (0.75% (*w*/*v*) proteose Peptone, 0.75% (*w*/*v*) Yeast extract, and 1.5% (*w*/*v*) Glucose) supplemented with 10 µg/mL gentamicin (Sigma-Aldrich, Madrid, Spain). As for the cytotoxicity assays, murine macrophages J774A.1 (ATCC TIB-67) were used, cultivated in DMEM medium (Dulbecco’s modified eagle medium, Gibco Life Technologies, California, USA) with 10% supplement of fetal bovine serum (FBS) and 10 µg/mL gentamicin at 37 °C in a 5% CO_2_ incubator.

Subsequently, DT was tested against two clinical isolates, *A.* *griffini*, genotype T3 obtained in a previous study [6] and *A. polyphaga* genotype T4 ATCC 30461. Those strains were grown axenically in the same medium as *A*. *castellanii* Neff.

As positive controls, chlorhexidine (chlorhexidine digluconate; Alfa Aesar, Thermo Fisher, Kandel, Germany), voriconazole (Sigma-Aldrich, Madrid, Spain), and amphotericin B (Sigma-Aldrich, Madrid, Spain) were used.

### 3.5. In Vitro Activity against Acanthamoeba spp. Trophozoites

The in vitro activities of the different compounds of *Laurencia* were evaluated using a previously [16] developed colorimetric assay based on the alamarBlue™ Cell Viability Reagent assay (Life Technologies, Barcelona, Spain). Briefly, 50 µL of 5 × 10⁴ trophozoites/mL were seeded onto a 96-well microtiter plate. After 15 min, the adherence of the cells to the plate bottom was confirmed by the use of the inverted microscope Leica DMIL (Leica, Wetzlar, Germany). Furthermore, 50 µL of serial dilutions of each compound was added to the 96-well microtiter plate. Finally, alamarBlue™ was placed into each well at 10% of the final volume and incubated at 26 °C with slight agitation. After 96 h, the fluorescence was measured with EnSpire Multimode Plate Reader (Perkin Elmer, Madrid, Spain) at a wavelength of 570/585 nm. The inhibitory concentration to inhibit the growth of 50% of the parasites (IC_50_) was calculated through nonlinear regression analysis with 95% confidence limits using the software Sigma Plot 12.0 (Systat Software Inc., London, UK). The experiments were performed three times each, allowing for standard deviation.

### 3.6. In vitro Activity against Acanthamoeba castellanii Neff cysts

The cysticidal activity of DT was determined by the alamarBlue™ assay and confirmed visually by inverted microscopy. Cysts of *A. castellanii* Neff were prepared as previously described [25]. Briefly, trophozoites were transferred from PYG medium based cultures (trophozoite medium) to Neff’s encystment medium (NEM; 0.1 M KCl, 8 mM MgSO_4_·7H_2_O, 0.4 mM CaCl_2_·2H_2_O, 1 mM NaHCO_3_, 20 mM ammediol (2-amino-2-methyl-1,3-propanediol; Sigma Aldrich, Madrid, Spain), pH 8.8 at 25 °C) and were cultured in this medium with gentle shaking for a week in order to obtain mature cysts. After that, mature cysts were harvested and washed twice using PYG medium.

A serial dilution of the tested molecules was made in PYG. The in vitro susceptibility assay was performed in sterile 96-well microtiter plates (Corning™). To these wells, containing 50 µL of drug dilutions, 5 × 10^4^ mature cysts of *Acanthamoeba*/mL were added. After 7 d of incubation with the drugs, the plate was centrifuged at 3000 rpm for 10 min. The supernatant was removed and replaced with 100 µL of fresh PYG medium in each well. Finally, 10 μL of the Alamar Blue Assay Reagent (Biosource, Europe, Nivelles, Belgium) was placed into each well, and the plates were then incubated for 144 h at 28 °C. The emitted fluorescence was periodically examined with an Enspire microplate reader (PerkinElmer, Waltham, MA, USA) at 570/585 nm.

### 3.7. Cytotoxicity Assay

The cytotoxicity of the tested compounds was determined in vitro with murine macrophages (J774A.1). For this assay, the DMEM culture medium was changed from RPMI without phenol red (Roswell Park Memorial Institute, Thermo Fisher Scientific Inc., Waltham, MA, USA) to L-Glutamine supplemented with 10% (*v*/*v*) inactivated FBS and with 10 µg/mL gentamicin. In this assay, the macrophages were counted and seeded into a 96-well microtiter plate, adding 50 µL to each well from a stock solution of 2 × 10^5^ cells/mL. Cells were then left to adhere for 15 min to the plate bottom and were checked with the inverted microscope Leica DMIL. After that, 50 µL of a serial dilution of the compounds was added to the 96-well microtiter plate. Finally, alamarBlue™ was added into each well at an amount equal to 10% of the final volume and incubated at 37 °C in the presence of CO_2_ at 5% for 24 h. After this period of time, as in the in vitro anti*-Acanthamoeba* assay, the fluorescence was determined with an EnSpire Multimode Plate Reader (PerkinElmer, Inc, Waltham, MA, USA) at a wavelength of 570/585 nm. The cytotoxicity concentration to inhibit the growth of 50% of murine macrophages (CC_50_) was calculated through nonlinear regression analysis with 95% confidence limits using the software Sigma Plot 12.0 (Systat Software, Inc, San Jose, CA 95110 USA). Experiments were performed three times each, allowing for standard deviation.

### 3.8. Double-Stain Assay for Programmed Cell Death Determination

A double-stain apoptosis detection kit (Hoechst 33342/PI) (GenScript, Piscataway, NJ, USA) and an EVOS FL Cell Imaging System AMF4300 (Life Technologies, Carlsbad, CA, USA) were used. The experiment was carried out by following the manufacturer’s recommendations, and 10^5^ cells/well were incubated in a 24-well plate for 24 h with the previously calculated IC_90_. The double-staining pattern allows the identification of three groups in a cellular population: live cells will show only a low level of fluorescence, cells undergoing programmed cell death (PCD) will show a higher level of blue fluorescence (as chromatin condenses), and dead cells will show low-blue and high-red fluorescence (as the propidium iodide stain enters the nucleus).

### 3.9. Intracellular Reactive Oxygen Species (ROS) Production Using CellROX^®^ Deep Red Staining

Generation of intracellular ROS was detected using the CellROX^®^ Deep Red fluorescent probe (Invitrogen, Thermo Fisher Scientific Inc., Waltham, MA, USA). The cells were treated with IC_90_ of DT for 24 h and exposed to CellROX^®^ Deep Red (5 μM, 30 min) at 26 °C in the dark. Cells were observed in a Leica TSC SPE confocal microscope equipped with inverted optics at λ_exc_ 633 and λ_em_ 519 nm.

### 3.10. Analysis of Mitochondrial Membrane Potential

Collapse of the electrochemical gradient across the mitochondrial membrane during apoptosis was detected with the JC-1 mitochondrial membrane potential detection kit (Cell Technology, Fremont, CA, USA). After being treated with IC_90_ of the test compound for 24 h, the cells were centrifuged (1000 rpm × 10 min) and resuspended in JC-1 buffer. Images were taken on an EVOS FL Cell Imaging System AMF4300 (Life Technologies, San Francisco, CA, USA) from Life Technologies (Madrid, Spain). The staining pattern allows the identification of two groups in a cellular population: live cells will show only red fluorescence; cells with low mitochondrial potential, (undergoing PCD) will show a higher level of green and red fluorescence.

### 3.11. Measurement of ATP Levels

The ATP level was measured using a Cell Titer-Glo^®^ Luminescent Cell Viability Assay (Promega, Alcobendas, Madrid). This required adding a single reagent (CellTiter-Glo^®^ Reagent, Promega, Alcobendas, Madrid), which relies on the properties of a proprietary thermostable luciferase, generating a stable proportional luminescent signal to the amount of ATP present, which is directly proportional to the number of cells present in culture. After incubation, treated amoebas were mixed with the kit reagent into a white-wall 96-well microtiter plate (Nunc; Thermo Fisher Scientific Inc., Waltham, MA, USA) following the manufacturer’s instructions for measurement of the luminescence on a PerkinElmer spectrophotometer. The effect of the drug on the ATP production was evaluated by incubating 10^5^ cells/mL with the previously calculated IC_90_ of DT following the manufacturer´s recommendations. As a negative control, an *A. castellanii* culture incubated with PYG medium was used.

### 3.12. Plasma Membrane Permeability

The SYTOX Green assay was performed to detect alterations of the membrane permeability in treated cells. Briefly, 10^5^ trophozoites were washed and incubated in saline solution with the SYTOX Green at a final concentration of 1 μM (Molecular Probes) for 15 min in the dark. Subsequently, DT solution was added (IC_90_). After 24 h of treatment, cells were observed in a Leica TSC SPE confocal microscope equipped with inverted optics at λ_exc_ 482 nm and λ_em_ 519 nm.

### 3.13. Statistical Analysis

All experiments were performed three times each in duplicate. The results obtained were analyzed using one-way ANOVA, and *p* values of <0.05 were considered significant. Statistical analyses were carried out using the GraphPad Prism8.0.2 software program (GraphPad Software, San Diego, CA, USA).

## 4. Conclusions

All tested oxasqualenoids molecules, with natural or semisynthetic origin, demonstrated activity against *Acanthamoeba castellanii* Neff trophozoites. Based on the in vitro amoebicidal activity and cytotoxicity assays, iubol (**3**) was the most active substance; it also turned out to be the most toxic against murine macrophages. The other active substance, the main metabolite dehydrothyrsiferol (**1**), was chosen as a scaffold for structure–activity relationship studies. The SAR analysis showed that the introduction of a hydroxyl group at C-15 (e.g., thyrsiferol (**2**)) or at C-22 (e.g., 22-hydroxydehydrothyrsiferol (**4**)) maintained the activity range but eliminated the toxic effects against macrophages, making them candidates for further activity and mechanisms of action studies**.** In addition, our studies using dehydrothyrsiferol (**1**) suggest that these substances cause inhibition of trophozoites by damages at the mitochondrial level. In this paper, we describe the activities against amoebae of a group of triterpenes produced by red algae of the genus *Laurencia*, establishing a new leader structure on which it is possible to design amoebicidal substances.

## Figures and Tables

**Table 1:** Effects of natural and semisynthetic oxasqualenoids (**1**–**16**) against *Acanthamoeba castellanii* Neff (IC_50_). Toxicity against murine macrophage J774.A1 (CC_50_). * Reference compounds.

Compound	IC_50_ (µM)	CC_50_ (µM)
Dehydrothyrsiferol (DT) (**1**)	12.83 ± 1.38 ^ab^	28.77 ±3.10 ^B^
Thyrsiferol (**2**)	13.97 ± 1.57 ^ab^	>100 ^D^
Iubol (**3**)	5.30 ± 0.87 ^ab^	7.72 ±0.22 ^A^
22-Hydroxydehydrothyrsiferol (**4**)	17.00 ± 4.57 ^ab^	>100 ^D^
1,2-Dehydropseudodehydrothyrsiferol (**5**)	104.76 ± 1.72 ^f^	>100 ^D^
Saiyacenol A (**6**)	55.43 ± 6.56 ^de^	59.91 ±8.50 ^C^
Saiyacenol B (**7**)	77.89 ± 3.30 ^ef^	>100 ^D^
28-Hydroxysaiyacenol A (**8**)	66.22 ± 3.81 ^de^	>100 ^D^
28-Hydroxysaiyacenol B (**9**)	59.92 ± 10.07 ^de^	>100 ^D^
Nivariol A (**10**)	101.70 ± 9.57 ^f^	>100 ^D^
Adejene B (**11**)	48.34 ± 0.55 ^cd^	>100 ^D^
18-Sulphatedehydrothyrsiferol (**12**)	43.18 ± 0.14 ^cd^	>100 ^D^
18-Ketodehydrothyrsiferol (**13**)	29.09 ± 2.84 ^bc^	23.37 ±1.76 ^B^
28-Iodosaiyacenol A (**14**)	50.46 ± 3.05 ^cd^	29.45 ±0.20 ^B^
28-Iodosaiyacenol B (**15**)	54.34 ± 6.56 ^de^	>100 ^D^
28-Hydroxythyrsiferol (**16**) ^1^	103.66 ± 8.42 ^f^	>100 ^D^
Chlorhexidine *	3.02 ± 0.89 ^a^	6.64 ±0.35 ^A^
Voriconazole *	0.94 ± 0.29 ^a^	2.64 ±0.27 ^A^
Amphotericin B *	39.65 ± 0.56 ^bcd^	>100 ^D^

^1^ These values correspond to a synthetic sample that contain a mixture of 15 *R/S* compounds. ^a–f^ The IC_50_ activity towards *Acanthamoeba* with different letters are significantly different with (*p* < 0.05). ^A–D^ The CC_50_ toxicity towards macrophages with different letters are significantly different (*p* < 0.05).
